# Effectiveness and safety of the bevacizumab and erlotinib combination *versus* erlotinib alone in EGFR mutant metastatic non-small-cell lung cancer: systematic review and meta-analysis

**DOI:** 10.3389/fonc.2023.1335373

**Published:** 2024-01-23

**Authors:** Rodrigo Motta-Guerrero, Alejandro Leon Garrido-Lecca, Virgilio E. Failoc-Rojas, Ana Calle-Villavicencio, Robert Villacorta-Carranza, Yesenia Huerta-Collado, Alicia Torres-Mera, Mario J. Valladares-Garrido, Víctor Rivera-Francia, Carlos Carracedo, Luis Raez

**Affiliations:** ^1^ALIADA Centro Oncologico, Lima, Peru; ^2^Universidad Cesar Vallejo, Piura, Peru; ^3^Universidad Nacional Pedro Ruiz Gallo, Lambayeque, Peru; ^4^Universidad Continental, Lima, Peru; ^5^Memorial Healthcare System, Florida, FL, United States

**Keywords:** non-small cell lung cancer, EGFR gene, VEGFR, tyrosine kinase inhibitor, erlotinib

## Abstract

**Background:**

The EGFR gene encodes a protein that stimulates molecular pathways that allow the growth and development of the tumor microenvironment. The current preferred tyrosine kinase inhibitor (TKI) for the first-line treatment of EGFRm metastatic non-small cell lung cancer (NSCLC) is osimertinib. However, the combination of angiogenesis inhibitors and TKI has produced discordant results. We aimed to assess the effects of the bevacizumab and erlotinib combination in EGFRm metastatic NSCLC.

**Methods:**

Using eligibility criteria focused on patients with EGFRm metastatic NSCLC treated with bevacizumab and erlotinib, we searched databases including clinical trial randomized studies and reviews published until April 15, 2023 in Medline (PubMed), Scopus, and Embase. Eight clinical trials (1,052 patients) were selected from 1,343 articles for quantitative and qualitative assessment. The risk of bias was assessed using the Cochrane Risk of Bias tool. Data were synthesized through random-effects meta-analysis.

**Results:**

The bevacizumab and erlotinib combination significantly improved the progression-free survival (PFS) (log(HR) = 0.63; 95% CI: 0.54–0.73, *p* < 0.001) and overall response ratio (ORR) (RR = 0.79; 95% CI, 0.64–0.97, *p* = 0.03). However, it did not improve the overall survival (log(HR) = 0.93; 95% CI, 0.78–1.10, *p* = 0.38) and was associated with higher serious adverse events (SAEs) (OR = 3.48; 95% CI, 1.76–6.88, *p* = 0.005). A subgroup analysis suggested similar benefits in different mutation subtypes and brain metastasis condition. The evidence is limited by a moderate risk of bias across studies and heterogeneity in the reporting of SAEs.

**Conclusions:**

The bevacizumab and erlotinib combination significantly improved PFS and ORR in EGFRm metastatic NSCLC but were also associated with higher-grade (≥3) adverse events. These results suggest that while the combination therapy may enhance progression-free survival and overall response, it does not improve the overall survival and is associated with higher toxicity. Thus, the treatment should be personalized based on individual patient comorbidities. Further prospective trials are needed to validate these results.

**Systematic review registration:**

https://www.crd.york.ac.uk/prospero/#searchadvanced, identifier CDR 42022364692.

## Introduction

According to GLOBOCAN, lung cancer has the second highest incidence worldwide, representing 11.4% of cases diagnosed with cancer. Lung cancer is also the leading cause of cancer death, with an estimated 1.8 million deaths in 2020 (1) . Tobacco smoking remains the predominant risk factor for lung cancer development (2). The epidermal growth factor receptor (EGFR) pathway is a well-studied oncogenic pathway in metastatic non-small cell lung cancer (NSCLC) (3) . The activation of the tyrosine kinase domain of the EGFR is a key reason for lung cancer progression (4) . The subsequent activation of the JAK-STAT, the PI3-K-Akt-mTOR, and the RAS-RAF-MEK-ERK pathways leads to cell proliferation, inhibition of apoptosis, and tumor microenvironment development (3, 5) . The prevalence of EGFR mutation is higher in younger non-smokers or light-smokers and those with wood smoke exposure (6) . The frequency of EGFR mutation varies widely worldwide and occurs more commonly (40%–60%) in the southeast of Asia (7) . It appears that Japan (64.8%), Thailand (57.8%), and Taiwan (54.3%) harbor the highest frequency of EGFR mutations in the Asian continent (8) . Meanwhile, the EGFR mutation rate in Western patients with adenocarcinoma is around 14%–19% (9) . In Latin America, it has been reported that Peru (51.1%), Mexico (34.3%), Costa Rica (31.4%), and Panama (27.3%) might harbor the highest rates (10) . EGFR mutant (EGFRm) metastatic non-small cell lung cancer (NSCLC) is generally sensitive to tyrosine kinase inhibitors (TKIs), considered the standard first line of treatment (11, 12) . TKIs have revolutionized the EGFRm metastatic NSCLC treatment landscape since the introduction of the first-generation TKIs as first-line treatment (6) . Second-generation and third-generation TKIs improved survival in comparison with the first-generation TKIs (13–17) . Osimertinib, a third-generation TKI, is the preferred agent for first-line therapy because of its significant central nervous system (CNS) activity and a favorable safety profile (11, 12, 18) . Different targets and regimens of treatment have been evaluated in EGFRm metastatic NSCLC. The vascular endothelial growth factor (VEGF) has been identified as a molecular pathway involved in the lung cancer tumoral microenvironment (3) . In the last decade, preclinical trials demonstrated that the combination of angiogenesis inhibitors and TKIs improves survival in EGFRm advanced NSCLC (19, 20) . However, discordant results limit its use in clinical practice (21–24) . The objective of this meta-analysis is to evaluate the safety and efficacy of the bevacizumab–erlotinib combination in EGFRm metastatic NSCLC.

## Materials and methods

### Study setting and eligibility criteria of studies

This systematic review was performed following the recommendations of the Cochrane Handbook for Systematic Reviews (25) , Preferred Reporting Items for Systematic Reviews and Meta-analyses (PRISMA) (26) , and AMSTAR 2 guidelines (27) . We previously registered the protocol in Prospective Register of Systematic Review (PROSPERO) (CDR 42022364692, registered on October 14, 2022).

We included studies that evaluated adults diagnosed with non-small cell lung cancer (NSCLC) by histological or cytological methods and who had a mutation in the epidermal growth factor receptor (EGFR). The participants were required to have a functional status score of 2 or lower, according to the criteria of the Eastern Cooperative Oncology Group (ECOG). Our review focused on randomized clinical trials (RCTs) that compared the combination of erlotinib and bevacizumab *versus* the use of erlotinib alone in the treatment of NSCLC with positive EGFR mutation. No restrictions were set in terms of race, gender, nationality, histological type, or smoking history of the participants. Studies that were reviews without primary data or extractable data, animal experiments or cadaver studies, patients with other types of cancer, patients who received previous treatment, and meta-analyses were excluded.

### Database and search strategy

We searched for clinical trial randomized studies published until April 15, 2023 in Medline (PubMed), Scopus, and Embase. We combined different keywords, controlled vocabulary terms (e.g., MeSH and Emtree terms), and free terms, according to the PICO strategy (population: “carcinoma, non-small-cell lung”; exposure: “erlotinib hydrochloride” AND “bevacizumab”; comparator: “erlotinib hydrochloride”) (**Supplementary Material**). The searches were not limited by date or language. We included articles in full text and excluded observational studies, review articles, abstracts, case reports, letters, editorials, studies not available in full text, and duplicated publications.

### Study selection and data extraction

We exported all retrieved references from databases to Rayyan QCRI (Rayyan Systems Inc.®, MA, USA). After removing duplicates, two authors (VEFR and ATM) performed independently the screening of title and abstracts. These authors independently reviewed the remaining references in the full text. Discrepancies were resolved by a third researcher. The references from retrieved papers were screened for additional articles. The articles found were analyzed using the terms of the PICO strategy and the inclusion and exclusion criteria. Relevant data from each article were extracted by two authors (VEFR and MJVG) independently and recorded in a spreadsheet of Microsoft Excel©: name of authors, year and country of publication, number of patients, number of events, and measure of association, with their 95% confidence intervals. Any conflict regarding the extracted information was resolved through consensus.

### Quality assessment

Two investigators (VEFR and ATM) independently evaluated the risk of bias in each eligible RCT. Any discrepancies were resolved by consensus or discussion with another investigator (MJVG). The Cochrane Collaboration tool for assessing the risk of bias in RCTs was used (28) . The following items were evaluated: generation of the allocation sequence (selection bias), concealment of the allocation sequence (selection bias), blinding (detection and performance bias), blinding of participants and personnel to outcome assessment, incomplete outcome data (attrition bias), selective outcome reporting (reporting bias), and other biases; each domain is a question and is answered according to yes/no/not known to rate it as high risk/low risk/unknowledge risk. Each RCT underwent a meticulous evaluation, with outcomes for each item categorized as having either a low risk of bias, a high risk of bias, or an unclear risk of bias.

### Outcome measures

Primary outcome variables were progression-free survival (PFS) defined as the time from randomization to tumor progression or death. Secondary outcomes were overall survival (OS) defined as the time from randomization to death, considered as the best therapeutic endpoint in cancer clinical trials, overall response ratio (ORR) defined as the proportion of patients whose symptoms were relieved to a predetermined value within the minimum time limit, and serious adverse event (SAE) defined as adverse event of grade 3 or more.

### Statistical analyses

In the meta-analysis, we pooled hazard ratios (HR) with 95% confidence intervals (95% CI) using fixed-effects models and followed the inverse variance method (due to the large number of events in each arm, more than 10%). The Paule–Mandel estimator was used for the assessment of the between-study variance (29) . Outcomes data available in ≥3 studies were meta-analyzed. Time-event variables, including OS and PFS, were assessed according to the HR. Dichotomous variables, including ORR and incidence of adverse events, were assessed as risk ratios (RR) with 95% confidence interval (CI) estimates. For studies reporting OR or RR stratified into different subgroups, we considered each subgroup analysis as a separate study. The quantitative synthesis was represented by forest plots. Heterogeneity among studies was assessed with Cochran’s Q test and Higgins *I*^2^ statistics; values of *I*^2^ <20%, 25%–50%, and >50% were defined as low, mild, and substantial heterogeneity, respectively. If *I*^2^ was <50% and *p* > 0.05, a fixed-effects model was used in the meta-analysis; if *I*^2^ ≥50% and *p* ≤ 0.05, a random-effects model was used to assess the resource of the heterogeneity. Publication bias was assessed with funnel plots and formally tested with Egger’s test.

All statistical analyses were conducted using RevMan version 5.4, provided by the Cochrane Collaboration. A *p*-value less than 0.05 was considered to indicate statistical significance.

## Results

### Study eligibility results

**Figure 1:**
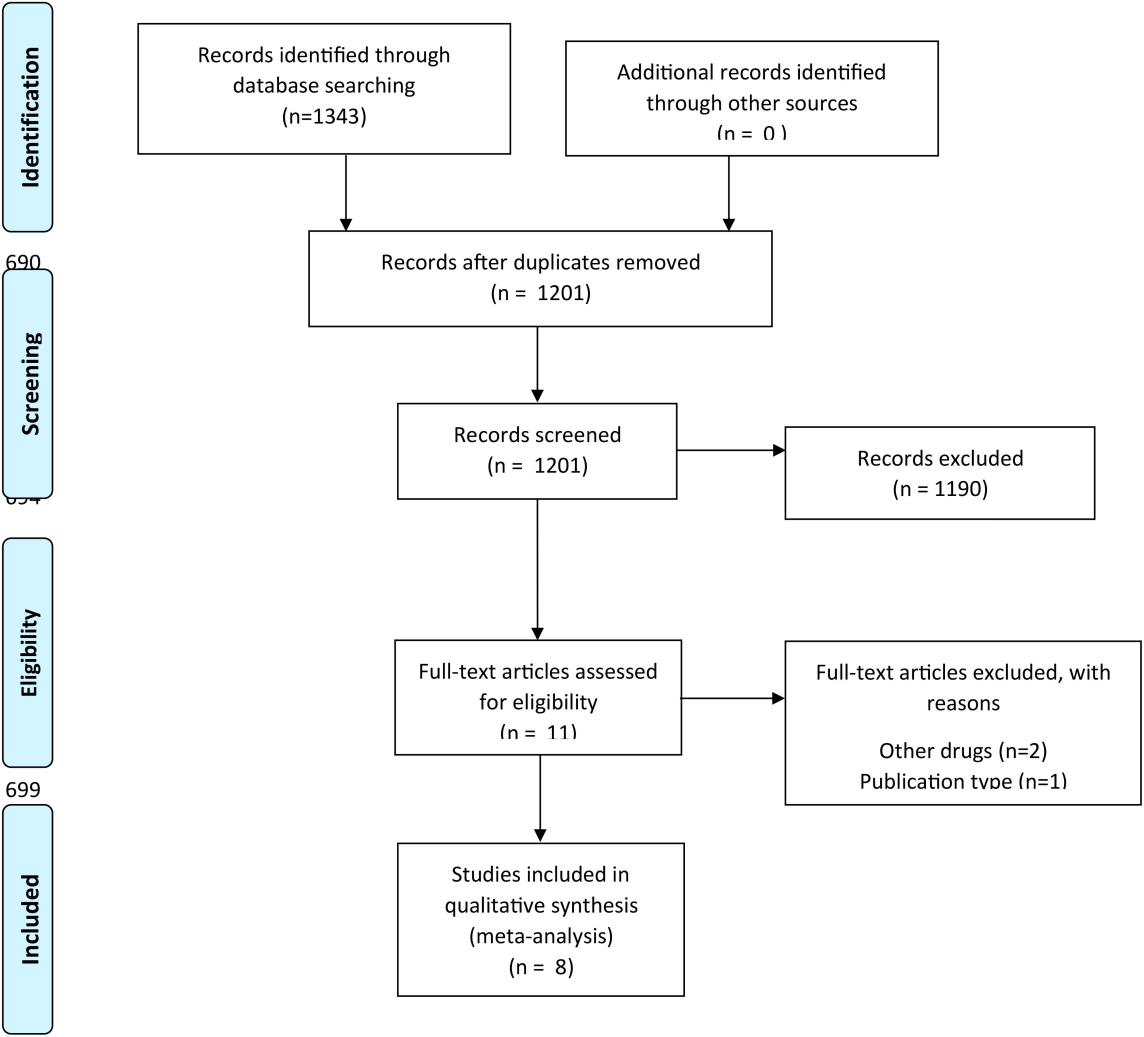
Flow diagram of the selection of reviews for analysis.

We collected a total of 1,343 articles in the primary search. After eliminating duplicates, 1,201 publications remained, which were evaluated in titles and abstracts. Subsequently, 11 articles that were analyzed in full text remained, of which eight clinical trials were selected for qualitative and quantitative assessment. The PRISMA checklist is provided in **Figure 1**. We only included full-text papers that reported adjusted association measures—HR—and a control group. The lack of a proper control group was the main cause for the exclusion of most studies (**Supplementary Material**).

### Study characteristics

This study included 1,052 patients with an age average range of 57–69 years and predominantly involving female patients. The included population was from China, Japan, Italy, South Korea, and the USA. Brain metastases were reported in 26%–47.6% of patients (**Table 1**).

**Table 1:** General characteristics of the studies included.

Study	Design, country	Patients	Age (mean or median)	Male (*n*, %)	Brain metastasis (*n*, %)	mPFS (95% IC)	mOS (95% IC)	ORR	Adverse events ≥3	Follow-up survival	Sponsor	NCT
**ARTEMIS-CTONG 1509 (2021)**	Phase III, China	Erlotinib + bevacizumab: 157	57	58 (37.7%)	47 (30.5%)	17.9 (15.2 to 19.9)	36.2 (32.5 to 42.4)	86.8%	54.80%	48 months	Guangdong Association of Clinical Trials	NCT02759614
		Erlotinib: 154	59	60 (38.2%)	44 (28.0%)	11.2 (9.7 to 13.8)	31.6 (27.2 to 40.0)	84.7%	26.10%			
**NEJ026** **Saito (** 30) Kawashima (2021)^a^	Phase III, Japan	Erlotinib + bevacizumab: 112	67	41 (37%)	36 (32%)	16.9 (14.2 to 21.0)	50.7 (37.3 to NE)	72%	88%	39.2 months	Chugai Pharmaceutical	UMIN000017069
		Erlotinib: 112	68	39 (35%)	36 (32%)	13.3 (11.1 to 15.3)	46.2 (38.2 to NE)	66%	46%			
**Stinchcombe et al. (** 21)	Phase II, USA	Erlotinib + bevacizumab: 43	65	12 (28%)	11 (26%)	17.9 (13.3 to 24.1)	32.4 (26.9 to 54.4)	81%	40%	33 months	Academic and Community Cancer Research United	NCT01532089
		Erlotinib: 45	63	14 (31%)	14 (31%)	13.5 (8.8 to 21.6)	50.6 (49.4 to NE)	83%	27%			
**BEVERLY (2022)**	Phase III, Italy	Erlotinib + bevacizumab: 80	65.9	28 (35%)	NA	14.7 (12.0 to 18.3)	33.3 (24.3 to 45.1)	NA	NA	36.3 months	National Cancer Institute, Naples	NCT02633189
		Erlotinib: 80	67.7	30 (37.5%)	NA	9.6 (7.1 to 10.6)	22.8 (18.3 to 33.0)	NA	NA			
**JO25567** **Seto (2014)** Yamamoto (2021)^a^	Phase II, Japan	Erlotinib + bevacizumab: 75	67	30 (40%)	NA	16.0 (13.9 to 18.1)	47	69%	91%	60 months	Chugai Pharmaceutical Co., Ltd.	JapicCTI-111390 (Japan)
		Erlotinib: 77	69	26 (34%)	NA	9.7 (5.7 to 11.1)	47.4	64%	53%			
**Lee et al. (** 31)	Phase II, South Korea	Erlotinib + bevacizumab: 64	NA	20 (31.2%)	29 (45.3%)	17.5 (12.5 to 22.5)	NA	85.9%	56.6%	38.9 months	National Cancer Center Research Grant	NCT03126799
		Erlotinib: 63	NA	23 (36.5%)	30 (47.6%)	12.4 (9.1 to 15.7)	NA	83.9%	20.6%			

a Used to evaluate overall survival.

mPFS, median progression-free survival; mOS, median overall survival; ORR, objective response rate; NCT, number of clinical trial.

### Meta-analysis of the effect of bevacizumab plus erlotinib on EGFR

#### Analysis of primary outcome

**Figure 2:**
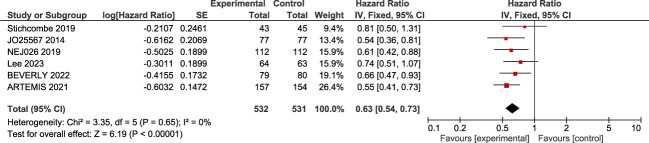
A forest plot of the effect of bevacizumab plus erlotinib on progression-free survival.

Six trials were included in analyzing bevacizumab and erlotinib in EGFRm metastatic NSCLC. A low heterogeneity among the six studies was found (*I*^2^ = 0%, *p* = 0.65). The result of the meta-analysis and the forest plot analysis showed that the bevacizumab and erlotinib combination improves progression-free survival in EGFRm advanced NSCLC, (log(HR) = 0.63; 95% CI: 0.54–0.73, *p* < 0.001) (**Figure 2**).

#### Analysis of secondary outcome (OS, ORR, and SAE)

##### Overall survival

**Figure 3:**
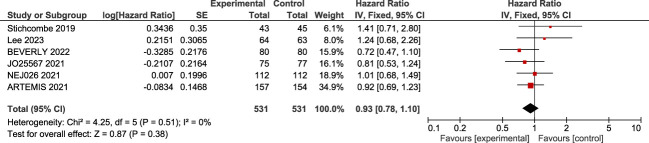
A forest plot of the effect of bevacizumab plus erlotinib on overall survival.

Six trials reported the median and confidence interval of overall survival as shown in the figure; the forest plot showed no significant enhancement in overall survival [log(HR) = 0.93; 95% CI, 0.78–1.10, *p* = 0.38]. There is no heterogeneity between the clinical trials (*I*^2^ = 0%; *p* = 0.51) (**Figure 3**).

##### Overall response ratio

**Figure 4:**
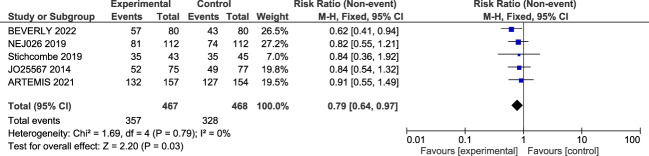
Forest plot of the secondary outcome; overall response rate (ORR).

The overall response ratio (ORR) was reported in five trials. The meta-analysis shows that a significant improvement in the overall response ratio was found (RR = 0.79; 95% confidence interval, 0.64–0.97, *p*-value = 0.03). Insignificant heterogeneity was detected among the studies (*I*^2^ = 0%, *p*-value = 0.79) (**Figure 4**).

##### Adverse events

**Figure 5:**
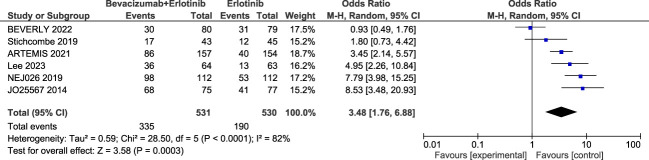
Forest plot of the secondary outcome; serious adverse event (SAE).

Serious adverse events (SAEs) were reported in six trials. The sub-group meta-analysis shows that SAEs are significantly higher with the combination (OR = 3.48; 95% confidence interval, 1.76–6.88, *p*-value <0.001), random effect. A highly significant heterogeneity was found among the studies (*I*^2^ = 82%, *p* < 0.0001) (**Figure 5**).

### Analysis by subgroups

A subgroup analysis was performed to assess whether the PFS varied by mutation, ECOG, and status. As shown in **Supplementary Figure S1**, the HR of the mutational group was similar in exon 19 deletion (HR = 0.62; 95% CI: 0.50–0.77) and exon 21 L858R (HR = 0.60; 95% CI: 0.47–0.77). This was similarly observed in ECOG 0 (HR = 0.61; 95% CI: 0.48–0.77) and ECOG 1 (HR = 0.62; 95% CI: 0.50–0.76). The report of three clinical trials (namely, ARTEMIS, Lee, and NEJ026) revealed that the combination bevacizumab + erlotinib resulted in a positive outcome for patients both with and without brain metastases, displaying a hazard ratio (HR) of 0.58 (95% CI: 0.41–0.81) and 0.63 (95% CI: 0.49–0.81), respectively. This is certainly an intriguing finding (**Supplementary Figure S3**). Only the BEVERLY trial reported a subgroup analysis of OS, so it was not possible to perform a subgroup meta-analysis for this outcome.

The grade ≥3 adverse events reported were diarrhea, hypertension, rash, and proteinuria. The risk of grade ≥3 diarrhea in the bevacizumab + erlotinib group was 53% higher than the risk of ≥3 diarrhea concerning erlotinib monotherapy (HR: 1.53; 95% CI: 0.82–2.86; *p* = 0.18). The risk of skin rash grade ≥3 was higher in the experimental group (HR: 1.49; 95% CI: 1.13–1.97; *I*^2^ = 0%). The risk of grade ≥3 hypertension in the erlotinib–bevacizumab group was found to be 5.1 times higher than that to the erlotinib group (HR = 5.10; 95% CI: 2.66–9.77; *I*^2^ = 56%). Finally, the combination also had a higher association of presenting grade ≥3 proteinuria than the erlotinib monotherapy group (HR = 12.33; 95% CI: 4.49–33.88; *I*^2^ = 0%). All forest plots can be found in **Supplementary Figure S4**.

### Risk of bias

The eight randomized clinical trials were analyzed, and a methodological review of Cochrane’s bias assessment was carried out, presenting the biases individually and as a group. Of the eight studies found, the BEVERLY trial showed a low risk of bias in all seven domains, followed by the studies of Lee et al. and Stinchcombe et al., which had a low risk of bias in the domains, except for the blinding of participants and personnel. The ARTEMIS trial presented biases in the blinding of participants and personnel as well as biases in the outcome assessors (as no information is mentioned in the protocol). In addition, the NEJ026 and the JO25567 trials presented other biases (due to pharmaceutical funding) or had unclear randomized methods (**Table 2**).

**Table 2:** Risk of individual bias of clinical trials that involve bevacizumab–erlotinib in first-line treatment of *EGFRm* advanced non-small cell lung cancer.

	Random sequence generation (selection bias)	Allocation concealment (selection bias)	Blinding of participants and personnel (performance bias)	Blinding of outcome assessment (detection bias)	Incomplete outcome data (attrition bias)	Selective reporting (reporting bias)	Other bias
ARTEMIS 2021	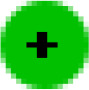	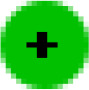	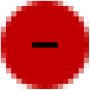	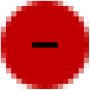	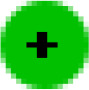	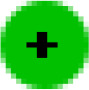	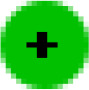
BEVERLY 2022	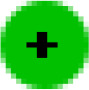	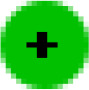	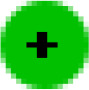	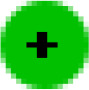	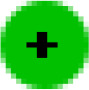	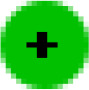	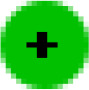
JO25567 2014	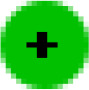	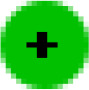	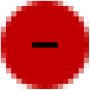	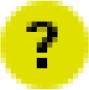	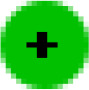	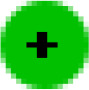	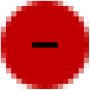
JO25567 2021	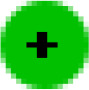	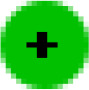	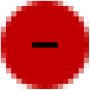	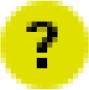	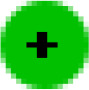	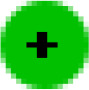	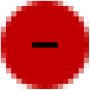
Lee 2023	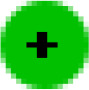	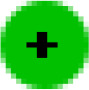	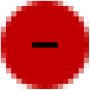	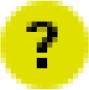	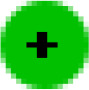	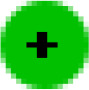	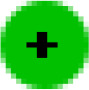
NEJ026 2019	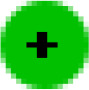	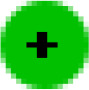	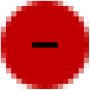	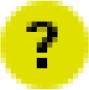	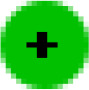	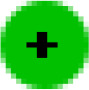	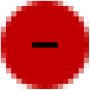
NEJ026 2021	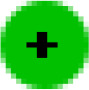	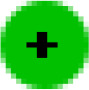	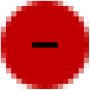	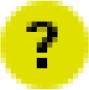	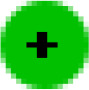	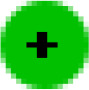	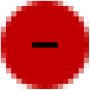
Stichcombe 2019	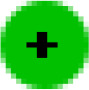	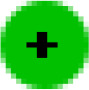	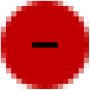	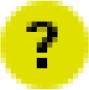	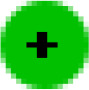	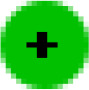	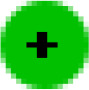

In general, the highest risk of bias was in the blinding of participants and personnel (open trials), followed by blinding of data assessors. All studies handled missing data well (intention-to-treat), as can be seen in **Table 3**. Despite the limitations presented, we are confident that the results obtained in each clinical trial are useful in terms of efficacy and safety.

**Table 3:** Bias assessment of the included primary studies.

	Random sequence generation (selection bias)								
	Allocation concealment (selection bias)								
	Blinding of participants and personnel (performance bias)								
	Blinding of outcome assessment (detection bias)								
	Incomplete outcome data (attrition bias)								
	Selective reporting (reporting bias)								
	Other bias								
		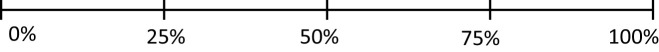							
	Low risk of bias								
	Unclear risk of bias								
	High risk of bias							

### Analysis of publication bias

**Figure 6:**
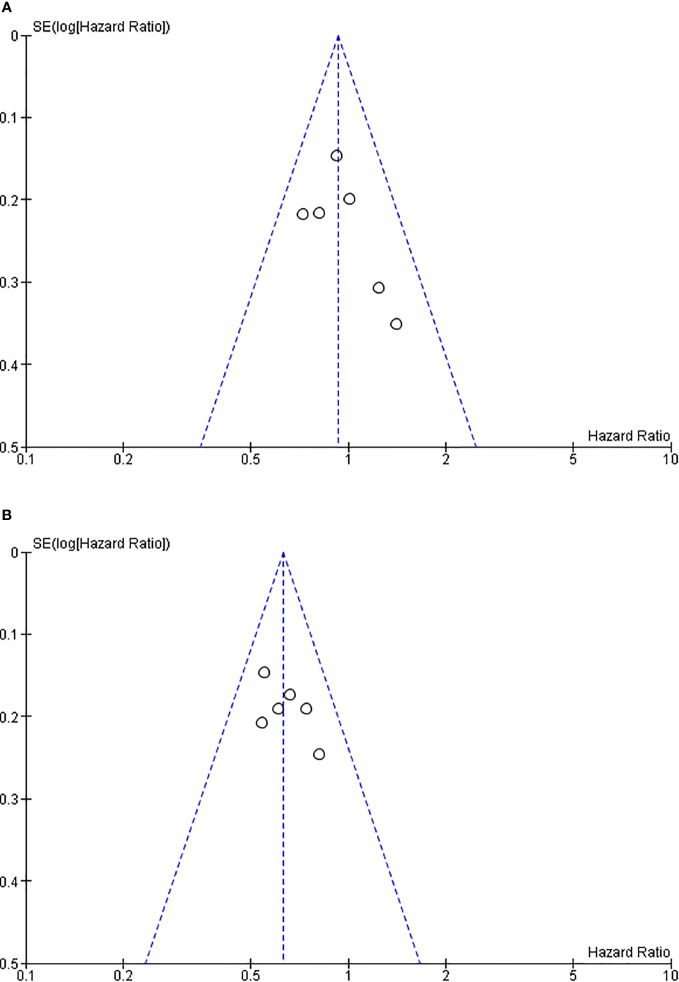
Funnel plot of clinical studies on erlotinib and bevacizumab. **(A)** Funnel plot of overall survival. **(B)** Funnel plot of progression-free survival.

The funnel plots for the studies included in the primary and secondary outcome are shown in **Figure 6**. A symmetric funnel plot was observed, with no evidence of publication bias among the studies. There was no evidence of apparent publication bias based on the assessment using a funnel plot and Egger’s test (*p* > 0.05).

## Discussion

This study provides new insights that could help resolve the controversies surrounding the combined use of erlotinib and bevacizumab in the treatment of EGFR-mutated NSCLC.

The EGFR gene encodes the protein located on the cell surface whose activation stimulates the molecular pathways that allow the growth and development of the tumor microenvironment (3, 5) . As previously described, osimertinib is the preferred TKI for the first line of treatment of EGFRm metastatic NSCLC (11, 12) . Several trials evaluated different combinations that could be safe and effective in this population, and the combination of angiogenesis inhibitors and TKI obtained discordant results (21–24) . Currently, osimertinib is the agent of choice for first-line treatment due to its greater penetrance in the CNS. However, the economic cost of osimertinib limits its access to clinical practice. An economic analysis reported that osimertinib as the first line of treatment is not cost-effective in high-income countries (32–34). Subsequent studies have evaluated possible combinations that could be options of therapy (3).

Our results show a statistically significant benefit in terms of progression-free survival (PFS) (HR = 0.63; 95% CI, 0.54–0.73). This result is consistent with those obtained in clinical trials and is similar to those obtained by other recently published meta-analyses (35–38) . The studies JO25567, ARTEMIS, NEJ026, and Stichcombe et al. obtained positive results in progression-free survival when evaluating the addition of an angiogenic inhibitor (bevacizumab) to the TKI (erlotinib) compared to a TKI given as a monodrug in first-line treatment for advanced NSCLC with EGFRm (9) . In 2014, one of the first clinical trials that was phase II (JO25567) showed that the addition of bevacizumab to erlotinib in patients with NSCLC improved PFS from 9.7 to 16.0 months in Japanese patients, with a HR of 0.54 (0.36 to 0.81). Another phase II clinical study (Stichcombe 2019) was the only study that did not report a statistical benefit of bevacizumab on PFS (HR: 0.81; 95% CI: 0.50–1.31); however, this study found a clinical benefit in mPFS of 17.9 vs. 13.5 months for the group of bevacizumab with erlotinib vs. erlotinib alone, respectively, similar to the phase II study by Lee et al. (2023) (31), which found no statistical benefit but observed a higher median PFS months (17.5 vs 12.4 months).

Another statistically significant result was ORR which was higher with the bevacizumab–erlotinib combination (RR = 0.79; 95% CI, 0.64–0.97, *p* = 0.03). However, higher ORR has not been replicated in all meta-analysis, probably because of heterogeneity (31, 35–40) . Our analysis only included clinical trials where the combination of bevacizumab and erlotinib was used to maintain a homogeneous population (*I*^2^ = 0%, *p* = 0.66).

Finally, no statistically significant benefit in overall survival (OS) was demonstrated (HR = 0.93; 95% CI, 0.78–1.10). It should be noted that the bevacizumab–erlotinib combination did not improve OS in clinical trials (21–24) . The consistent lack of benefit in OS could be explained by the subsequent line of treatment. Patients with progression of disease received osimertinib as second line when T790M mutation was detected in blood or tumoral tissue. Patients were treated with osimertinib after progression of disease: 29.2%, 57.1%, and 43% of patients in the erlotinib group and 17.2%, 49%, and 45% of patients in the bevacizumab–erlotinib group in the ARTEMIS, BEVERLY, and NEJ026 trials, respectively (21, 30, 41) . Based on the details above, we believe that there could be a methodological limitation that affects the accuracy of the OS results; this could affect the accuracy of OS results due to the difference in the intensity of subsequent therapy between the two groups.

SAEs were more common with bevacizumab–erlotinib combination. However, despite the higher toxicity, clinical trials conclude that bevacizumab–erlotinib is safe, with manageable toxicity (21–23) . Another meta-analysis indicates that the angiogenesis inhibitors and TKI combination is safe in NSCLC patients (38, 42) . Using the combination in first-line therapy may lead to the sequency of treatment with osimertinib in second-line. Clinical trials report a similar prevalence of T790M mutation after first-generation TKIs and the combination progression of disease. In addition, an economic analysis reported that osimertinib is cost-effective in second-line (43, 44).

In a subgroup analysis, the results indicate that the bevacizumab–erlotinib combination is associated with longer PFS in exon 19 deletion or exon 21 L858R mutations, suggesting that this combination may be similarly effective in both molecular subtypes. This result is consistent with that obtained in a recently published meta-analysis, suggesting that L858R mutations may be as much benefited as exon 19 deletion (38) . Further investigation is needed to conclude if this combination of treatment is the best option in L858R mutations.

The presence of brain metastasis is a recognized adverse prognostic factor and a frequent site of disease progression in EGFR-mutated NSCLC. Previous research has shown that the anti-VEGF medication bevacizumab is a successful treatment for brain metastases and can also significantly decrease their incidence (45).

Bevacizumab has demonstrated a significant clinical benefit in CNS tumors, highlighting its anti-edema effect. This effect is particularly relevant in the reduction of post-radiation brain edema, a common complication in patients with brain metastases (46, 47); in addition, bevacizumab has shown a beneficial effect in the treatment of refractory brain edema with an efficacy rate of 84.74% (46). Bevacizumab improves vascular permeability and reduces fluid infiltration into brain tissue, which helps mitigate the adverse effects of edema and improves treatment efficacy (47). This mechanism may explain the improvement in progression-free survival observed in patients with brain metastases following a combination treatment with erlotinib and bevacizumab.

Grade ≥3 AEs were more commonly reported with bevacizumab–erlotinib combination (diarrhea, rash, hypertension, and proteinuria). The grade ≥3 toxicity described is mainly secondary to bevacizumab. The toxicity of the combination therapy should be considered according to the patient’s comorbidities when making treatment choices. Patients with chronic hypertension that is not controlled or chronic kidney disease may not be candidates for the bevacizumab–erlotinib combination. In addition, the implementation of a strict monitoring program of blood pressure measurement and urinalysis in patients receiving bevacizumab must be done when treating these patients.

Recent studies have highlighted significant advances in predicting treatment response in advanced NSCLC. On the one hand, research has shown that changes in tumor density and volume measured by CBCT during therapy can correlate with clinical outcomes, offering a promising method for predicting treatment response at an early stage. On the other hand, radiomic signatures based on pre-treatment CT images have demonstrated their ability to predict PD-L1 expression and TMB status, and these signatures can be combined with clinical and morphological factors to improve the predictive efficacy and targeting of immunotherapy (48, 49).

Recently conducted clinical trials on osimertinib and bevacizumab have failed to demonstrate any improvement in either PFS or OS as compared to osimertinib alone among patients suffering from NSCLC with an EGFR mutation, regardless of whether they are undergoing first-line (24) or second-line treatment (50). Despite the existence of only phase II studies, the potential for combination therapy appears to be unpromising.

The enhanced PFS value observed when utilizing erlotinib-plus-bevacizumab may be attributed to modifications in tumor vessel physiology caused by bevacizumab, ultimately leading to greater intratumoral drug uptake and improved drug delivery (51).

As we have indicated above, without considering aspects of economic studies, third-generation TKIs (osimertinib) are considered as first-line treatment in some regions of the world based on the analysis of multiple measures for second-line treatment after drug resistance. However, we believe that some patients may miss the therapeutic opportunity to receive treatment with third-generation TKIs due to failure to detect the presence of the T790M mutation. The FLAURA trial showed that first-line osimertinib is similar to second-line osimertinib and still has a very beneficial effect (17), but more evidence is needed to determine the most effective and reasonable treatment line in the use of osimertinib. Accordingly, we believe that the combination of erlotinib + bevacizumab may be very useful in those countries where osimertinib is not cost-effective (32–34) or in patients where the T790M mutation has not been detected (or it is not possible to sequence).

A recently published systematic review and meta-analysis by Sakharkar et al. (2023) (52) compared the efficacy and safety of the combination of erlotinib and bevacizumab with erlotinib monotherapy in patients with advanced first- or second-line NSCLC. The combination of erlotinib plus bevacizumab significantly prolonged PFS (HR: 0.62, 95% CI: 0.56–0.70, *p* < 0.001) but did not show a significant improvement in OS (HR: 0.95, 95% CI: 0.83–1.07, *p* = 0.39) and ORR (HR: 1.03, 95% CI: 1.02–1.07, *p* = 0.01) and ORR (HR: 1.10; 95% CI: 0.98–1.24, *p* = 0.09). Our results report a similar HR in PFS, but slightly lower in OS, and our results (evaluated in the first-line setting) found a benefit of the combination in ORR (RR: 0.79; *p* = 0.003). A network meta-analysis (53) aimed at comparing different first-line regimens for NSCLC treatment in an Asian population found that the highest scoring regimens were erlotinib (Erl) plus Bev (SUCRA: 0.94) and Erl plus ramucirumab (Ram) (SUCRA: 0.93) (but only one study) compared to bevacizumab alone (SUCRA: 0.87). These results, which are like those found in our study, suggest that while there are benefits in terms of PFS, the combination may carry additional risks that should be considered but can be considered as a good therapeutic option. We found similar hazard ratios (HRs) for both exon 19 deletions and exon 21 L858R mutations. This suggests that the combination therapy may be effective across different EGFR mutation subtypes, offering a potentially valuable treatment option for patients with these common EGFR mutations. Given the distinct molecular pathways activated by different EGFR mutations, our findings provide a basis for tailoring treatment approaches based on specific mutation profiles.

Based on the details above, we believe that, in clinical practice, a patient who is unable to use osimertinib and must instead receive erlotinib may benefit from the combination with bevacizumab, given the presence of exon 19 or exon 21 mutation, brain metastasis, and no contraindication of anti-angiogenic. Regarding future research directions, it is essential to conduct further studies to evaluate the effectiveness and safety of different combinations and sequences of TKIs in advanced NSCLC. Ongoing clinical trials (NCT02856893 and NCT03790397) are examining the optimal sequence of gefitinib and osimertinib in first-line treatment, which may provide insight into the best treatment strategy. This combination could be particularly beneficial for patients with brain metastases, given bevacizumab’s demonstrated effectiveness in treating CNS tumors and reducing post-radiation brain edema. However, it is crucial to consider individual patient factors such as comorbidities and potential for adverse events when selecting this combination therapy. Our study highlights the need for accessible and effective treatment options in regions with limited resources and underscores the importance of expanding global access to a broader range of therapies for NSCLC.

The present meta-analysis has limitations that should be considered. Firstly, the quantitative synthesis comprised six studies, some with little sample sizes, phases of clinical trials, and follow-up periods as previously stated. This heterogeneity may affect the results. Secondly, the meta-analysis was conducted at the trial level rather than the individual patient level. A sensitivity analysis was infeasible due to a limited number of eligible studies, many of which were open-label. Therefore, potential prognostic factors, patient comorbidities, extent of disease, and other genetic mutations were not examined in our study, potentially constraining our analyses. Despite the study’s limitations, the rigorous process of selecting studies enabled a comprehensive evaluation of the available evidence related to the topic of interest. We adhered to the PRISMA, Cochrane, and PROSPERO guidelines.

In conclusion, the study shows that the bevacizumab–erlotinib combination significantly improves PFS and ORR in EGFRm metastatic NSCLC. Bevacizumab–erlotinib is also associated with higher grade ≥3 adverse events. Although toxicity is manageable, a patient’s comorbidities must be strongly considered when using treatment with the bevacizumab–erlotinib combination. In addition, the combination may be an option in the first-line in countries without access to osimertinib. Prospective trials are needed to validate the benefit in L585R mutations.

## Data availability statement

The data analyzed in this study is subject to the following licenses/restrictions: The data is free to obtain from the selected clinical studies; if you wish to have a copy, you can ask the corresponding author with a justification by mail. Requests to access these datasets should be directed to VF-R (virgiliofr@gmail.com).

## Author contributions

RM-G: Conceptualization, Investigation, Writing – original draft, Writing – review & editing. ALG-L: Conceptualization, Writing – original draft, Writing – review & editing, Supervision, Validation. VF-R: Conceptualization, Writing – original draft, Writing – review & editing, Data curation, Formal Analysis, Methodology. AC-V: Conceptualization, Validation, Writing – original draft, Writing – review & editing. RV-C: Conceptualization, Methodology, Writing – original draft, Writing – review & editing. YH-C: Investigation, Visualization, Writing – original draft, Writing – review & editing. AT: Conceptualization, Data curation, Formal Analysis, Writing – original draft, Writing – review & editing. MV-G: Conceptualization, Investigation, Methodology, Validation, Writing – original draft, Writing – review & editing. VR-F: Conceptualization, Investigation, Supervision, Writing – original draft, Writing – review & editing. CC: Conceptualization, Supervision, Validation, Writing – original draft, Writing – review & editing. LR: Supervision, Validation, Writing – original draft, Writing – review & editing.

## References

[1] Sung H Ferlay J Siegel RL Laversanne M Soerjomataram I Jemal A . Global cancer statistics 2020: GLOBOCAN estimates of incidence and mortality worldwide for 36 cancers in 185 countries. CA: Cancer J Clin (2021) 71(3):209–49. doi: 10.3322/caac.21660 33538338

[2] Bade BC Dela Cruz CS . Lung cancer 2020: epidemiology, etiology, and prevention. Clinics chest Med (2020) 41(1):1–24. doi: 10.1016/j.ccm.2019.10.001 32008623

[3] Hsu PC Jablons DM Yang CT You L . Epidermal growth factor receptor (EGFR) pathway, yes-associated protein (YAP) and the regulation of programmed death-ligand 1 (PD-L1) in non-small cell lung cancer (NSCLC). Int J Mol Sci (2019) 20(15):3821. doi: 10.3390/ijms20153821 31387256 PMC6695603

[4] Liu TC Jin X Wang Y Wang K . Role of epidermal growth factor receptor in lung cancer and targeted therapies. Am J Cancer Res (2017) 7(2):187–202. 28337370 PMC5336495

[5] Siegelin MD Borczuk AC . Epidermal growth factor receptor mutations in lung adenocarcinoma. Lab investigation J Tech Methods Pathol (2014) 94(2):129–37. doi: 10.1038/labinvest.2013.147 24378644

[6] Heredia D Mas L Cardona AF Oyervides V Motta Guerrero R Galvez-Nino M . A high number of co-occurring genomic alterations detected by NGS is associated with worse clinical outcomes in advanced EGFR-mutant lung adenocarcinoma: Data from LATAM population. Lung Cancer (Amsterdam Netherlands) (2022) 174:133–40. doi: 10.1016/j.lungcan.2022.11.002 36379126

[7] Hsu WH Yang JC Mok TS Loong HH . Overview of current systemic management of EGFR-mutant NSCLC. Ann Oncol (2018) 29(suppl_1):i3–9. doi: 10.1093/annonc/mdx702 29462253

[8] Yatabe Y Kerr KM Utomo A Rajadurai P Tran VK Du X . EGFR mutation testing practices within the Asia Pacific region: results of a multicenter diagnostic survey. J Thorac Oncol (2015) 10(3):438–45. doi: 10.1097/jto.0000000000000422 PMC4342317 25376513

[9] Han B Tjulandin S Hagiwara K Normanno N Wulandari L Laktionov K . EGFR mutation prevalence in Asia-Pacific and Russian patients with advanced NSCLC of adenocarcinoma and non-adenocarcinoma histology: The IGNITE study. Lung Cancer (Amsterdam Netherlands) (2017) 113:37–44. doi: 10.1016/j.lungcan.2017.08.021 29110846

[10] Arrieta O Cardona AF Martín C Más-López L Corrales-Rodríguez L Bramuglia G . Updated frequency of EGFR and KRAS mutations in nonSmall-cell lung cancer in Latin America: the Latin-American consortium for the investigation of lung cancer (CLICaP). J Thorac Oncol (2015) 10(5):838–43. doi: 10.1097/jto.0000000000000481 25634006

[11] Ettinger DS Wood DE Aisner DL Akerley W Bauman JR Bharat A . Non-small cell lung cancer, version 3.2022, NCCN clinical practice guidelines in oncology. J Natl Compr Cancer Network: JNCCN (2022) 20(5):497–530. doi: 10.6004/jnccn.2022.0025 35545176

[12] Hendriks LE Kerr KM Menis J Mok TS Nestle U Passaro A . Non-oncogene-addicted metastatic non-small-cell lung cancer: ESMO Clinical Practice Guideline for diagnosis, treatment and follow-up. Ann Oncol (2023) 34(4):358–76. doi: 10.1016/j.annonc.2022.12.013 36669645

[13] Wu YL Cheng Y Zhou X Lee KH Nakagawa K Niho S . Dacomitinib versus gefitinib as first-line treatment for patients with EGFR-mutation-positive non-small-cell lung cancer (ARCHER 1050): a randomised, open-label, phase 3 trial. Lancet Oncol (2017) 18(11):1454–66. doi: 10.1016/s1470-2045(17)30608-3 28958502

[14] Yang JC Hirsh V Schuler M Yamamoto N O'Byrne KJ Mok TS . Symptom control and quality of life in LUX-Lung 3: a phase III study of afatinib or cisplatin/pemetrexed in patients with advanced lung adenocarcinoma with EGFR mutations. J Clin Oncol (2013) 31(27):3342–50. doi: 10.1200/jco.2012.46.1764 23816967

[15] Mok TS Cheng Y Zhou X Lee KH Nakagawa K Niho S . Updated overall survival in a randomized study comparing dacomitinib with gefitinib as first-line treatment in patients with advanced non-small-cell lung cancer and EGFR-activating mutations. Drugs (2021) 81(2):257–66. doi: 10.1007/s40265-020-01441-6 PMC7932969 33331989

[16] Zhou C Wu YL Chen G Feng J Liu XQ Wang C . Final overall survival results from a randomised, phase III study of erlotinib versus chemotherapy as first-line treatment of EGFR mutation-positive advanced non-small-cell lung cancer (OPTIMAL, CTONG-0802). Ann Oncol (2015) 26(9):1877–83. doi: 10.1093/annonc/mdv276 26141208

[17] Ramalingam SS Vansteenkiste J Planchard D . Overall survival with osimertinib in untreated, EGFR-mutated advanced NSCLC. New Engl J Med (2020) 382(1):41–50. doi: 10.1056/NEJMoa1913662 31751012

[18] Lazzari C Gregorc V Karachaliou N Rosell R Santarpia M . Mechanisms of resistance to osimertinib. J Thorac Dis (2020) 12(5):2851–8. doi: 10.21037/jtd.2019.08.30 PMC7330330 32642198

[19] Boussageon M Swalduz A Pérol M . The safety and efficacy of erlotinib and ramucirumab combination in EGFR-mutant non-small-cell lung cancer. Expert Rev Anticancer Ther (2021) 21(10):1071–80. doi: 10.1080/14737140.2021.1958679 34281470

[20] Ninomiya T Takigawa N Ichihara E Ochi N Murakami T Honda Y . Afatinib prolongs survival compared with gefitinib in an epidermal growth factor receptor-driven lung cancer model. Mol Cancer Ther (2013) 12(5):589–97. doi: 10.1158/1535-7163.mct-12-0885 23443806

[21] Stinchcombe TE Jänne PA Wang X Bertino EM Weiss J Bazhenova L . Effect of erlotinib plus bevacizumab vs erlotinib alone on progression-free survival in patients with advanced EGFR-mutant non-small cell lung cancer: A phase 2 randomized clinical trial. JAMA Oncol (2019) 5(10):1448–55. doi: 10.1001/jamaoncol.2019.1847 PMC6692685 31393548

[22] Nakagawa K Garon EB Seto T Nishio M Ponce Aix S Paz-Ares L . Ramucirumab plus erlotinib in patients with untreated, EGFR-mutated, advanced non-small-cell lung cancer (RELAY): a randomised, double-blind, placebo-controlled, phase 3 trial. Lancet Oncol (2019) 20(12):1655–69. doi: 10.1016/s1470-2045(19)30634-5 31591063

[23] Zhou Q Xu CR Cheng Y Liu YP Chen GY Cui JW . Bevacizumab plus erlotinib in Chinese patients with untreated, EGFR-mutated, advanced NSCLC (ARTEMIS-CTONG1509): A multicenter phase 3 study. Cancer Cell (2021) 39(9):1279–1291.e3. doi: 10.1016/j.ccell.2021.07.005 34388377

[24] Kenmotsu H Wakuda K Mori K Kato T Sugawara S Kirita K . Randomized phase 2 study of osimertinib plus bevacizumab versus osimertinib for untreated patients with nonsquamous NSCLC harboring EGFR mutations: WJOG9717L study. J Thorac Oncol (2022) 17(9):1098–108. doi: 10.1016/j.jtho.2022.05.006 35636696

[25] Green S Higgins J . Cochrane handbook for systematic reviews of interventions version 5.1. 0 Vol. 2009. The Cochrane Collaboration (2011).

[26] Moher D Liberati A Tetzlaff J Altman DG . Preferred reporting items for systematic reviews and meta-analyses: the PRISMA statement. Ann Internal Med (2009) 151(4):264–9, w64. doi: 10.7326/0003-4819-151-4-200908180-00135 19622511

[27] Shea BJ Reeves BC Wells G Thuku M Hamel C Moran J . AMSTAR 2: a critical appraisal tool for systematic reviews that include randomised or non-randomised studies of healthcare interventions, or both. bmj (2017) 358:j4008. doi: 10.1136/bmj.j4008 28935701 PMC5833365

[28] Cumpston M Li T Page MJ Chandler J Welch VA Higgins JP . Updated guidance for trusted systematic reviews: a new edition of the Cochrane Handbook for Systematic Reviews of Interventions. Cochrane Database systematic Rev (2019) 10:Ed000142. doi: 10.1002/14651858.ed000142 PMC10284251 31643080

[29] van Aert RCM Jackson D . Multistep estimators of the between-study variance: The relationship with the Paule-Mandel estimator. Stat Med (2018) 37(17):2616–29. doi: 10.1002/sim.7665 PMC6055723 29700839

[30] Saito H Fukuhara T Furuya N Watanabe K Sugawara S Iwasawa S . Erlotinib plus bevacizumab versus erlotinib alone in patients with EGFR-positive advanced non-squamous non-small-cell lung cancer (NEJ026): interim analysis of an open-label, randomised, multicentre, phase 3 trial. Lancet Oncol (2019) 20(5):625–35. doi: 10.1016/s1470-2045(19)30035-x 30975627

[31] Lee Y Kim HR Hong MH Lee KH Park KU Lee GK . A randomized Phase 2 study to compare erlotinib with or without bevacizumab in previously untreated patients with advanced non-small cell lung cancer with EGFR mutation. Cancer (2023) 129(3):405–14. doi: 10.1002/cncr.34553 PMC10100207 36451343

[32] Shu Y Ding Y He X Liu Y Wu P Zhang Q . Cost-effectiveness of osimertinib versus standard EGFR-TKI as first-line treatment for EGFR-mutated advanced non-small-cell lung cancer in China. Front Pharmacol (2022) 13:920479. doi: 10.3389/fphar.2022.920479 36204237 PMC9531913

[33] Aguiar PN Jr Haaland B Park W San Tan P Del Giglio A de Lima Lopes G Jr. Cost-effectiveness of osimertinib in the first-line treatment of patients with EGFR-mutated advanced non-small cell lung cancer. JAMA Oncol (2018) 4(8):1080–4. doi: 10.1001/jamaoncol.2018.1395 PMC6143050 29852038

[34] Aguilar-Serra J Gimeno-Ballester V Pastor-Clerigues A Milara J Marti-Bonmati E Trigo-Vicente C . Osimertinib in first-line treatment of advanced EGFR-mutated non-small-cell lung cancer: a cost-effectiveness analysis. J Comp Eff Res (2019) 8(11):853–63. doi: 10.2217/cer-2019-0029 31478399

[35] Chen F Chen N Yu Y Cui J . Efficacy and safety of epidermal growth factor receptor (EGFR) inhibitors plus antiangiogenic agents as first-line treatments for patients with advanced EGFR-mutated non-small cell lung cancer: A meta-analysis. Front Oncol (2020) 10:904. doi: 10.3389/fonc.2020.00904 32714857 PMC7344312

[36] Yang Y Wang L Li X Zhang S Yu J Nie X . Efficacy and safety of bevacizumab combined with EGFR-TKIs in advanced non-small cell lung cancer: A meta-analysis. Thorac cancer Jan (2022) 13(1):31–7. doi: 10.1111/1759-7714.14214 PMC8720617 34859599

[37] Sun L Ma JT Zhang SL Zou HW Han CB . Efficacy and safety of chemotherapy or tyrosine kinase inhibitors combined with bevacizumab versus chemotherapy or tyrosine kinase inhibitors alone in the treatment of non-small cell lung cancer: a systematic review and meta-analysis. Med Oncol (Northwood London England) (2015) 32(2):473. doi: 10.1007/s12032-014-0473-y 25603953

[38] Zhang S Li S Liu J Yang C Zhang L Bao H . Comparative efficacy and safety of TKIs alone or in combination with antiangiogenic agents in advanced EGFR-mutated NSCLC as the first-line treatment: A systematic review and meta-analysis. Clin Lung Cancer (2022) 23(2):159–69. doi: 10.1016/j.cllc.2021.06.001 34247986

[39] Chen Z Wei J Ma X Yu J . Efficacy of EGFR-TKIs with or without angiogenesis inhibitors in advanced non-small-cell lung cancer: A systematic review and meta-analysis. J Cancer (2020) 11(3):686–95. doi: 10.7150/jca.34957 PMC6959046 31942192

[40] Ma JT Guo YJ Song J Sun L Zhang SL Huang LT . Rational application of first-line EGFR-TKIs combined with antiangiogenic inhibitors in advanced EGFR-mutant non-small-cell lung cancer: A systematic review and meta-analysis. BioMed Res Int (2021) 2021:8850256. doi: 10.1155/2021/8850256 33575349 PMC7861921

[41] Piccirillo MC Bonanno L Garassino MC Esposito G Dazzi C Cavanna L . Addition of bevacizumab to erlotinib as first-line treatment of patients with EGFR-mutated advanced nonsquamous NSCLC: the BEVERLY multicenter randomized phase 3 trial. J Thorac Oncol (2022) 17(9):1086–97. doi: 10.1016/j.jtho.2022.05.008 35659580

[42] Xiao YY Zhan P Yuan DM Lv TF Song Y Shi Y . Chemotherapy plus multitargeted antiangiogenic tyrosine kinase inhibitors or chemotherapy alone in advanced NSCLC: a meta-analysis of randomized controlled trials. Eur J Clin Pharmacol (2013) 69(2):151–9. doi: 10.1007/s00228-012-1333-3 22729611

[43] Guan H Liu G Xie F Sheng Y Shi L . Cost-effectiveness of osimertinib as a second-line treatment in patients with EGFR-mutated advanced non-small cell lung cancer in China. Clin Ther (2019) 41(11):2308–2320.e11. doi: 10.1016/j.clinthera.2019.09.008 31607559

[44] Bertranou E Bodnar C Dansk V Greystoke A Large S Dyer M . Cost-effectiveness of osimertinib in the UK for advanced EGFR-T790M non-small cell lung cancer. J Med Econ (2018) 21(2):113–21. doi: 10.1080/13696998.2017.1377718 28880737

[45] Masuda C Sugimoto M Wakita D Monnai M Ishimaru C Nakamura R . Bevacizumab suppresses the growth of established non-small-cell lung cancer brain metastases in a hematogenous brain metastasis model. Clin Exp Metastasis (2020) 37(1):199–207. doi: 10.1007/s10585-019-10008-z 31768815 PMC7007905

[46] Meng X Zhao R Shen G Dong D Ding L Wu S . Efficacy and safety of bevacizumab treatment for refractory brain edema: Case report. Med (Baltimore) (2017) 96(44):e8280. doi: 10.1097/MD.0000000000008280 PMC5682776 29095257

[47] Hua YC Gao DZ Wang KY Ding XS Xu WR Li YB . Bevacizumab reduces peritumoral brain edema in lung cancer brain metastases after radiotherapy. Thorac Cancer (2023) 14(31):3133–9. doi: 10.1111/1759-7714.15106 PMC10626225 37718465

[48] Wen Q Zhu J Meng X Ma C Bai T Sun X . The value of CBCT-based tumor density and volume variations in prediction of early response to chemoradiation therapy in advanced NSCLC. Sci Rep (2017) 7(1):14650. doi: 10.1038/s41598-017-14548-w 29116100 PMC5676710

[49] Wen Q Yang Z Dai H Feng A Li Q . Radiomics study for predicting the expression of PD-L1 and tumor mutation burden in non-small cell lung cancer based on CT images and clinicopathological features. Front Oncol (2021) 11:620246. doi: 10.3389/fonc.2021.620246 34422625 PMC8377473

[50] Soo RA Han JY Dafni U Cho BC Yeo CM Nadal E . A randomised phase II study of osimertinib and bevacizumab versus osimertinib alone as second-line targeted treatment in advanced NSCLC with confirmed EGFR and acquired T790M mutations: the European Thoracic Oncology Platform (ETOP 10-16) BOOSTER trial. Ann Oncol (2022) 33(2):181–92. doi: 10.1016/j.annonc.2021.11.010 34839016

[51] Manzo A Montanino A Carillio G Costanzo R Sandomenico C Normanno N . Angiogenesis inhibitors in NSCLC. Int J Mol Sci (2017) 18(10):2021. doi: 10.3390/ijms18102021 28934120 PMC5666703

[52] Sakharkar P Kurup S . Comparing efficacy of erlotinib and bevacizumab combination with erlotinib monotherapy in patients with advanced non-small cell lung cancer (NSCLC): A systematic review and meta-analysis. Diseases (2023) 11(4):146. doi: 10.3390/diseases11040146 37873790 PMC10594499

[53] Chen W Miao J Wang Y Xing W Xu X Wu R . Comparison of the efficacy and safety of first-line treatments for of advanced EGFR mutation-positive non-small-cell lung cancer in Asian populations: a systematic review and network meta-analysis. Front Pharmacol (2023) 14:1212313. doi: 10.3389/fphar.2023.1212313 37484016 PMC10358853

