# Supplementary material for: Effectiveness and safety of the bevacizumab and erlotinib combination versus erlotinib alone in EGFR mutant metastatic non-small-cell lung cancer: systematic review and meta-analysis
Source: Front Oncol. 2024 Jan 23;13:1335373. doi: 10.3389/fonc.2023.1335373 (PMC10846309; doi:10.3389/fonc.2023.1335373)

**SEARCH STRATEGY**

**Table S1: Search strategy for erlotinib and bevacizumab in patients with non-small cell lung cancer.**

| **Search** | **Search strategy (15/04/2023)** |
| --- | --- |
| PUBMED |  |
| #1 | (NSCLC[Tiab] OR (("Carcinoma, Non-Small-Cell Lung"[Mesh] OR "Non-Small Cell lung"[Tiab] OR "Non-Small-Cell lung"[Tiab] OR "Non-Small Cell lung"[Tiab]) AND (Neoplas*[Tiab] OR Carcinoma[TIAB] OR Cancer*[TIAB] OR Malignan*[Tiab]))) |
| #2 | ("Erlotinib Hydrochloride"[Mesh] OR "erlotinib"[Tiab] OR DA87705X9K) |
| #3 | (anti-VEGF[Tiab] OR "anti VEGF"[Tiab] OR "Bevacizumab"[Mesh] OR "Bevacizumab"[Tiab] OR 2S9ZZM9Q9V OR Avastin[Tiab] OR Tarceva[Tiab]) |
| #4 | (("Randomized Controlled Trial" [Publication Type]) OR ("Controlled Clinical Trial" [Publication Type]) OR ("Double-Blind Method"[Mesh] OR "Random Allocation"[Mesh] OR "Single-Blind Method"[Mesh]) OR (“Clinical trials as Topic”[Mesh]) OR ("Clinical Trial" [Publication Type])) |
| #1 AND #2 AND #3 AND #4 | 198 |
| EMBASE | |
| #1 | (NSCLC:ti,ab OR (('Carcinoma, Non-Small-Cell Lung'/exp OR 'Non-Small Cell lung':ti,ab OR 'Non-Small-Cell lung':ti,ab OR 'Non-Small Cell lung':ti,ab) AND (Neoplas*:ti,ab OR Carcinoma:ti,ab OR Cancer*:ti,ab OR Malignan*:ti,ab))) |
| #2 | ('Erlotinib Hydrochloride'/exp OR erlotinib:ti,ab OR DA87705X9K |
| #3 | (anti-VEGF:ti,ab OR 'anti VEGF':ti,ab OR Bevacizumab/exp OR Bevacizumab:ti,ab OR 2S9ZZM9Q9V OR Avastin:ti,ab OR Tarceva:ti,ab) |
| #4 | 'Double-Blind Method'/exp OR 'Random Allocation'/exp OR 'Single-Blind Method'/exp OR 'Clinical trials as Topic'/exp OR (random*:ti,ab) |
| #1 AND #2 AND #3 AND #4 | 1099 |
| SCOPUS | |
| #1 | (TITLE-ABS(NSCLC) OR ((INDEXTERMS("Carcinoma, Non-Small-Cell Lung") OR TITLE-ABS("Non-Small Cell lung") OR TITLE-ABS("Non-Small-Cell lung") OR TITLE-ABS("Non-Small Cell lung")) |
| #2 | (TITLE-ABS(Neoplas*) OR TITLE-ABS(Carcinoma) OR TITLE-ABS(Cancer*) OR TITLE-ABS(Malignan*)))) |
| #3 | (INDEXTERMS("Erlotinib Hydrochloride") OR TITLE-ABS(erlotinib) OR DA87705X9K ) AND (TITLE-ABS(anti-VEGF) OR TITLE-ABS("anti VEGF") OR INDEXTERMS(Bevacizumab) OR TITLE-ABS(Bevacizumab) OR 2S9ZZM9Q9V OR TITLE-ABS(Avastin) OR TITLE-ABS(Tarceva)) |
| #4 | (DOCTYPE("Randomized Controlled Trial") OR DOCTYPE("Controlled Clinical Trial") OR INDEXTERMS("Double-Blind Method") OR INDEXTERMS("Random Allocation") OR INDEXTERMS("Single-Blind Method") OR INDEXTERMS("Clinical trials as Topic") OR DOCTYPE("Clinical Trial")) |
| #1 AND #2 AND #3 AND #4 | 46 |

**Table S2 *-*** General characteristics of the studies excluded.

| Study, year, country | Title | Reason for exclusion |
| --- | --- | --- |
| Zhou Q, 2019, China. | CTONG 1509: Phase III study of bevacizumab with or without erlotinib in untreated Chinese patients with advanced EGFR-mutated NSCLC | Type of publication, abstract. |
| Rosell R, 2017, International multicentre. | Erlotinib and bevacizumab in patients with advanced non-small-cell lung cancer and activating EGFR mutations (BELIEF): an international, multicentre, single-arm, phase 2 trial | No group control. |
| Nakagawa, 2019, Multicentre. | Ramucirumab plus erlotinib in patients with untreated, EGFR-mutated, advanced non-small-cell lung cancer (RELAY): a randomised, double-blind, placebo-controlled, phase 3 trial. | Other drug, Ramucirumab |

**Figure S1**: Forest plot of progression-free disease-free survival according to EGFR mutation in NSCLC with EGFR mutations.


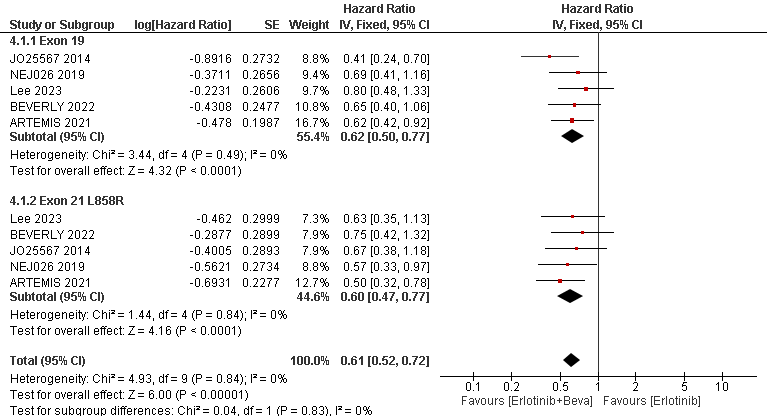


**Figure S2**: Forest plot of progression-free disease-free survival according to ECOG in NSCLC with EGFR mutations.


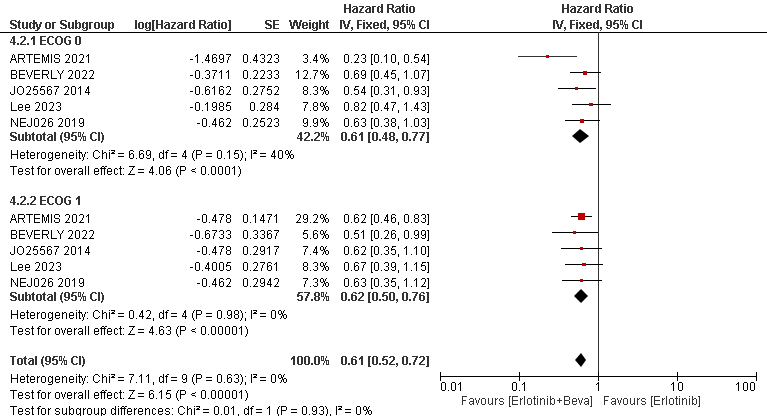


**Figure S3**: Forest plot of progression-free disease-free survival according to brain metastasis in NSCLC with EGFR mutations.


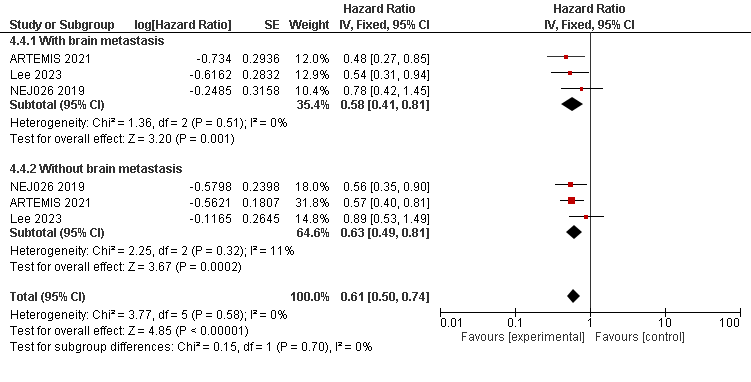


**Figure S4**: Forest plot of adverse events occurring in 3 or more in NSCLC with EGFR mutations.

1. Diarrhea (EA 3 or more)


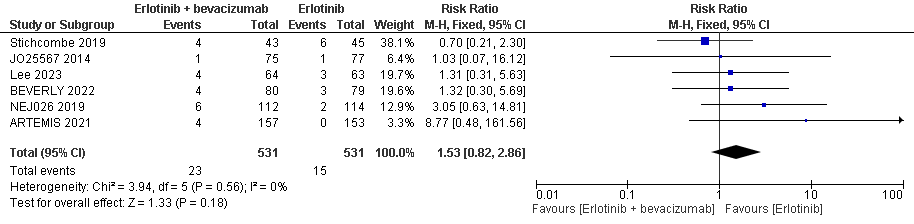


1. Hypertension (EA 3 or more)


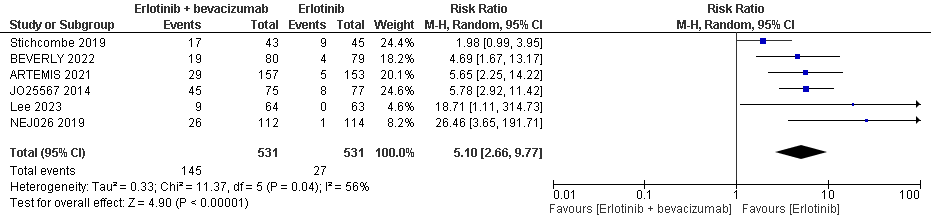


1. Proteinuria (EA 3 or more)


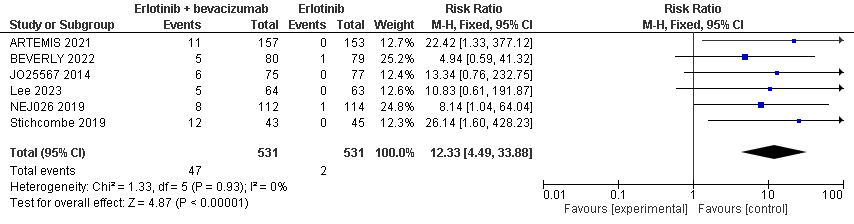


1. Rash (EA 3 or more)


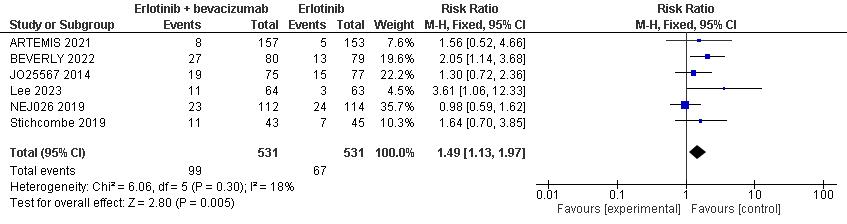

Supplement: Supplementary file 1 [file DataSheet_1.docx]
